# Catalytic valorization of hardwood for enhanced xylose-hydrolysate recovery and cellulose enzymatic efficiency via synergistic effect of Fe^3+^ and acetic acid

**DOI:** 10.1186/s13068-019-1587-4

**Published:** 2019-10-17

**Authors:** Kaixuan Huang, Lalitendu Das, Jianming Guo, Yong Xu

**Affiliations:** 1 Key Laboratory of Forestry Genetics & Biotechnology (Nanjing Forestry University), Ministry of Education, Nanjing, 210037 People’s Republic of China; 2 Jiangsu Co-Innovation Center of Efficient Processing and Utilization of Forest Resources, Jiangsu Province Key Laboratory of Green Biomass-based Fuels and Chemicals, College of Chemical Engineering, Nanjing Forestry University, Nanjing, 210037 People’s Republic of China; 3 Joint BioEnergy Institute, 5885 Hollis Street, Emeryville, CA 94608 USA; 4 Biomass Science and Conversion Technology, Sandia National Laboratories, 7011 East Avenue, Livermore, CA 94551 USA

**Keywords:** Poplar, Acetic acid catalysis, Fe^3+^-assisted hydrolysis, Xylose-hydrolysate recovery, Enzymatic saccharification of cellulose

## Abstract

**Background:**

Poplars are considered suitable dedicated energy crops, with abundant cellulose and hemicellulose, and huge surplus biomass potential in China. Xylan, the major hemicellulosic component, contributes to the structural stability of wood and represents a tremendous quantity of biobased chemicals for fuel production. Monomeric xylose conversion to value-added chemicals such as furfural, xylitol, and xylonic acid could greatly improve the economics of pulp-paper industry and biorefinery. Acetic acid (HAc) is used as a friendly and recyclable selective catalyst amenable to xylan degradation and xylooligosaccharides production from lignocellulosic materials. However, HAc catalyst usually works much feebly at inert woods than agricultural straws. In this study, effects of different iron species in HAc media on poplar xylan degradation were systematically compared, and a preferable Fe^3+^-assisted HAc hydrolysis process was proposed for comparable xylose-hydrolysate recovery (XHR) and enzymatic saccharification of cellulose.

**Results:**

In presence of 6.5% HAc with 0.17–0.25 wt% Fe^3+^, xylose yield ranged between 72.5 and 73.9%. Additionally, pretreatment was effective in poplar delignification, with a lignin yield falling between 38.6 and 42.5%. Under similar conditions, saccharification efficiency varied between 60.3 and 65.9%. Starting with 100 g poplar biomass, a total amount of 12.7–12.8 g of xylose and 18.8–22.8 g of glucose were harvested from liquid streams during the whole process of Fe^3+^-HAc hydrolysis coupled with enzymatic saccharification. Furthermore, the enhancement mechanism of Fe^3+^ coupled with HAc was investigated after proof-of-concept experiments. Beechwood xylan and xylose were treated under the same condition as poplar sawdust fractionation, giving understanding of the effect of catalysts on the hydrolysis pathway from wood xylan to xylose and furfural by Fe^3+^-HAc.

**Conclusions:**

The Fe^3+^-assisted HAc hydrolysis process was demonstrated as an effective approach to the wood xylose and other monosaccharides production. Synergistic effect of Lewis acid site and aqueous acetic acid provided a promising strategy for catalytic valorization of poplar biomass.
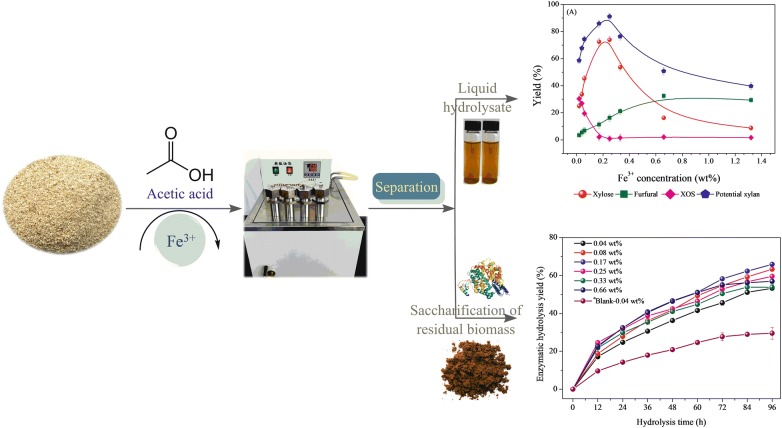

## Background

Plant biomass has been suggested as an abundant and sustainable source for the future chemicals, fuels, and materials due to its carbon-neutral nature and renewability [1, 2]. Biomass valorization has drawn interest in recent years for processing lignocellulosic fractions via selective hydrolysis routes to release valuable platform chemicals [3]. Poplar sawdust, a wood waste residue after felling, cutting and processing by the furniture factory, is one of the surplus forest residues in China. In general, the wood residues are favorable biomass sources for their superior properties such as high calorific value, low ash content and slagging rate [4]. However, the highly heterogeneous structure can be resistant to effective fractionation into fermentable sugars. Hence, a pretreatment strategy to fractionate poplar sawdust has become a crucial unit operation, to increase the enzymatic digestibility of cellulose whilst leaving out lignin for other economic purposes [5].

Poplar xylan consists of a linear xylose polymer as a backbone with a relatively high content of *O*-acetyl substitutions and 4-*O*-methyl α-d-glucosyluronic acid in the hemicelluloses fractions making it susceptible to the thermal hydrolysis [1, 6]. Hence, the hydrolysis of poplar-associated xylan primarily produces the pentosan sugar xylose. This xylose can be gathered and co-fermented with glucose to further boost bioethanol production [7]. Alternatively, monomeric xylose can be employed as a feedstock via dehydration, hydrogenation or biological routes to produce other commercial products such as furfural, xylitol, and xylonic acid, which have huge market potential [8–10]. Currently, hydrolyzing poplar xylan to maximize xylan degradation (or xylose production) along with efficient enzymatic saccharification remains a problem, because under acidic conditions furfural can be continuously stripped out from pentose sugar [11–14]. An optimized and controllable hydrolysis reaction would successfully facilitate decomposition of xylan, which renders glucan vulnerable to enzymatic hydrolysis. Maximizing the production of xylose acquired from the acidic media of poplar xylan is a priority both for enhancing the capability of enzymatic hydrolysis and as a means of capitalizing on valuable commercial xylose products.

Among acidic pretreatment methods, acetic acid (HAc) a recyclable and cheap catalyst is as effective as mineral acids [14, 15]. Besides, acetic acid is cost-effective, safe to operate and easy to recover, especially being capable of high-efficient recycling by a simple vacuum distillation, which can yield over 75% recovery from the crude biomass hydrolysate and a yield up to 98% acetic acid recovery could be further realized by the formation of azeotrope (water-HAc) [15]. Our previous work on poplar and corncob biomass illustrated the efficacy of HAc in converting xylan to xylooligosaccharides (XOS). Additionally, we found HAc was effective in releasing glucose from corncob, while it was ineffective in achieving higher glucose yield from poplar due to its recalcitrance nature [11, 16]. Studies on HAc biomass pretreatment focusing on the xylose production report temperatures between 100 and 150 °C as optimal operating condition [17–20]. Nonetheless, in the case of poplar relatively high temperatures of pretreatment were implemented for xylan degradation resulting in a reduction of the cellulose crystallinity, which has been demonstrated by our previous study [11]. During the catalytic reaction, the hydronium ions in acids promote the cleavage of glycosidic bonds present in the xylan, liberating free xylose that can be detected in the liquid phase. However, the occurrence of undesired parallel reactions can also take place, which are involved in secondary losses such as the dehydration of pentoses to furfural, along with the subsequent condensation (e.g., humins), resinification (e.g., resinous products) or decomposition [21–24]. To this end, monitoring the hydrolytic reaction in situ to minimize the formation of furfural is of great importance.

In addition, many acidic heterogeneous catalysts have been involved in biomass conversion. Recently, Mao et al. [14] developed a seawater-based furfural process co-existing with HAc steam and FeCl_3_, resulting in 72.9% furfural production and 79.5% lignin removal. For a more efficient enzymatic hydrolysis, the effects of FeCl_3_, CuCl_2_, and AlCl_3_ on the pretreated corn stover have been assessed. Results suggest that metal chloride salts display superior performance than H_2_SO_4_ at the same reaction condition [25]. Commonly, various acids including Lewis acids can be used to increase the hydrolytic reactivity during the acid hydrothermal fractionation (low pH) [1]. Besides, ions such as Fe^3+^ and Al^3+^ showed stronger cation exchange capacity than H^+^, being capable of coordinating to the oxygen atoms of the carbohydrate and enhancing the removal of xylan during pretreatment, leading in sugar gains for enzymatic digestibility [26–28]. Among reported catalysts, FeCl_3_ is preferably selected for grass/herb pretreatment, such as corncob, corn stover, and rice straw owing its nontoxic, low-cost and high selective nature [29–32]. Due to the strong acidity and oxidative power (*E*° = 0.77 V for Fe^3+^/Fe^2+^), the FeCl_3_-catalyzed biomass conversion process could be a promising technology that needs further investigation [2]. Moreover, Ogura et al. [33] achieved 90% of the theoretical ethanol yield during the fermentation by *Saccharomyces cerevisiae* after pretreatment with the [BMIM] Cl/HCl + FeCl_3_ (4.8 wt%) + acetone–water system and subsequent saccharification, which suggests that enough water washing on pretreated biomass would eliminate the detrimental effects of soluble catalysts and lignocellulose-derived inhibitors on microbial growth. Additionally, it is expected to recover ferric chloride by adjusting pH with sodium hydroxide until the formation of ferric hydroxide precipitation. Then vacuum filtration could be used to separate and recover ferric hydroxide solid. Furthermore, the recovered ferric chloride can participate in Fenton reaction for the disposal of wastewater [34]. However, so far there have been limited studies combining inorganic salts in the aqueous acetic acid pretreatment of hardwood (e.g., poplar). The presence of higher lignin content creates hindrance for the effective dissolution of lignocellulosic biomass [35]. For this reason, implementation of metal salts and HAc in poplar xylan hydrolysis shows potential in enhancing hemicellulose sugar recovery and enzymatic efficiency of cellulose.

In this study, we report an integrated approach to use HAc as aqueous reaction media employing Fe ions as catalyst to explore a sustainable alternative for the effective recovery of hemicellulosic sugar (mainly xylose) from poplar and for promoting glucan recovery and delignification. Specifically, the Fe ions-assisted HAc fractionation was designed for efficient degradation of poplar xylan and release of xylose into the hydrolysate for further downstream processing. First, the effects of different Fe species (Fe^2+^, Fe^3+^, Fe^8/3+^) on poplar xylan degradation were investigated. In addition, we systematically investigated the combined effect of Fe^3+^ and HAc on xylose production, glucan recovery, and lignin removal. Based on experimental data, a plausible mechanism of Fe^3+^ and HAc stimulating the xylose production was proposed.

## Results and discussion

### Influence of different iron species on poplar xylan degradation in aqueous HAc media

This section was designed to investigate the effectiveness of different Fe species on poplar xylan degradation in the presence of HAc solution. To further examine the fact that Fe ions could increase the xylan degradation, three different Fe salts, including trivalent Fe salt FeCl_3_, divalent FeSO_4_, and iron oxide Fe_3_O_4_ were explored. To eliminate the interference of Fe ion concentration levels, the Fe ion concentrations were selected in the range of 0.02–0.08 wt% based on FeSO_4_, Fe_3_O_4_, and FeCl_3_, respectively.

**Fig. 1:**
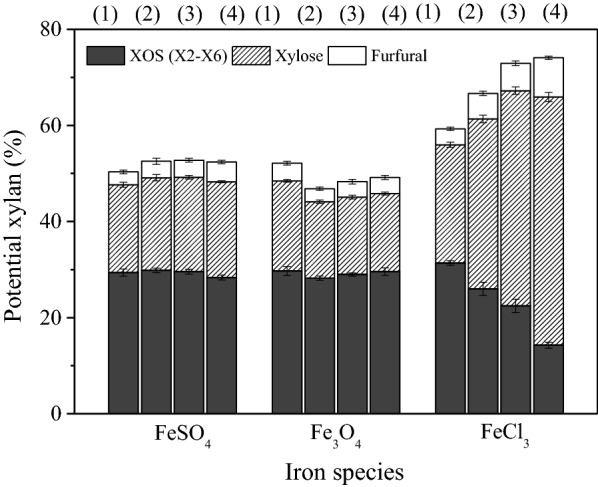
Effects of various iron species, i.e., FeSO_4_, Fe_3_O_4_ and FeCl_3_, on production of XOS (X2–X6), xylose and furfural in aqueous HAc media. (all concentration in wt%, unless otherwise stated) (1) 0.02 wt%; (2) 0.04 wt%; (3) 0.06 wt%; (4) 0.08 wt% of Fe^2+^, Fe^8/3+^, and Fe^3+^

The reaction was carried out with 6.5% HAc solution at a temperature of 170 °C. The fate of poplar xylan was determined by quantifying the yield of XOS (X2–X6), xylose and furfural. As shown in Fig. 1, in the cases of FeSO_4_ and Fe_3_O_4_, increasing the concentration of Fe ions made little contribution to xylan degradation to chemicals (XOS, xylose, and furfural). By contrast, the potential xylan was found to continuously increase from 58.7 to 75.0% as [Fe^3+^] increased from 0.02 to 0.08 wt%, indicating that high concentrations of FeCl_3_ caused more xylan conversion. FeCl_3_ exerted different effects on XOS, xylose, and furfural production during the hydrolysis process. For instance, it was observed that XOS yields declined from 31.0 to 14.4%, while the generated xylose and furfural increased from 24.2% and 3.4% to 52.3% and 8.3%, respectively, after being treated by the elevated concentration of Fe^3+^ salt. These results suggest that Fe^3+^ could promote the decomposition of xylan and further degrade oligosaccharides into monosaccharides and other by-products, such as furfural [1, 36]. Overall result indicate that Fe^3+^ salt exhibit better performance on wood xylan degradation than that of Fe^2+^ and Fe^8/3+^ salts, which is consistent with the result related to corn stover pretreatment by inorganic salts [32]. Notably, Yan et al. [37] also reported that Cl^−^ exerted stronger effects on the hemicelluloses degradation, indicating the interaction behavior or combination between salts cations and anions were eye-catching that cannot be ignored.

### Influence of Fe^3+^ concentration on xylose-hydrolysate recovery (XHR)

**Fig. 2:**
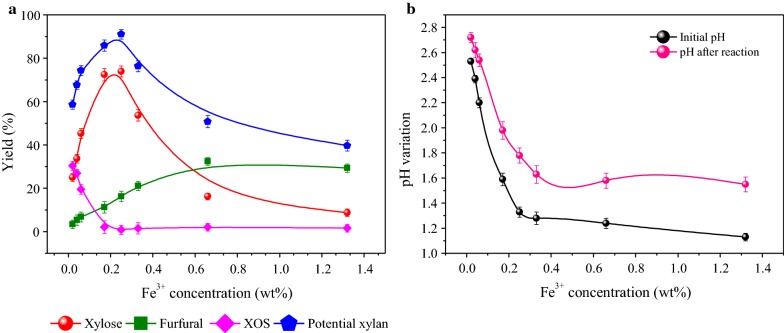
Process optimization of sugar release after Fe^3+^-assisted HAc pretreatment. **a** Effects of Fe^3+^ concentration on the fate of hemicellulose in aqueous HAc media pretreated at 170 °C for 27 min. **b** pH variations as a function of added Fe^3+^ concentrations in the presence of HAc prior to and immediately after the pretreatment

The influence of [Fe^3+^] ranging from 0.02 to 1.32 wt% on poplar xylan degradation was also investigated with a concentration of 6.5% HAc. As illustrated in Fig. 2a, the impacts of Fe^3+^ as an enhancer of HAc on xylose production, generating by-products XOS and furfural during xylan conversion process were rigorously examined. It was observed that xylose yield increased from 25.0 to 73.9% as [Fe^3+^] ratio increased from 0.02 to 0.25 wt%, but a further increase of [Fe^3+^] to 1.32 wt% decreased the yield (8.8%). Similarly, the potential xylan showed an increasing trend, then decreasing with the maximum value of 91.1% at 0.25 wt% of [Fe^3+^], indicating faster rate of xylose degradation than the production rate of furfural. Meanwhile, when [Fe^3+^] reached 0.25 wt%, XOS decreased to zero, indicating that [Fe^3+^] could accelerate the decomposition of dissolved oligomeric xylose into monomeric xylose [32]. In the same way, the observed decrease in xylose yield demonstrated that increasing [Fe^3+^] ratio favored the xylose formation, but an excessive Fe^3+^ resulted in more degradation products derived from monomeric xylose. Additionally, furfural yield dropped from 32.5 to 29.3% as [Fe^3+^] increased from 0.66 to 1.32 wt%. Liu and Wyman [38] also reported that the dosage of 0.8% FeCl_3_ could accelerate furfural resinification and condensation. Similarly, Mao et al. [39] found that the catalysts FeCl_3_ and HAc co-catalyzing hydrolysis of corncob promoted the formation of furfural (67.9%) but paid less attention to extracting xylose. Taken together, results illustrate that at the optimal concentrations of 0.17 wt% and 0.25 wt% [Fe^3+^], HAc media enabled XHR generate maximum xylose yields of 72.5% and 73.9%, respectively.

pH can be described as an indicator of the stability of a hydrolysis system, as it depends on the buffering capacity of the biomass or reaction itself [40]. When different concentrations of Fe^3+^ salt were added (0.02–1.32 wt%) to the HAc media, pH variations as a function of [Fe^3+^] prior to and immediately after the pretreatment were also recorded. The pH value corresponding to each added Fe^3+^ concentration is displayed in Fig. 2b. It was observed that consistent pH decreasing trend (*∆* = 1.4 pH units) occurred as [Fe^3+^] increased from 0.02 to 1.32 wt%, indicating the presence of Fe^3+^ could contribute an enhancement in the acid strength of the aqueous acetic acid solution. Notably, the highest xylose production can be obtained at the initial pH value around 1.3 corresponding to 0.25 wt% concentration of Fe^3+^ which has proven to be preferable for the effective pretreatment. Nonetheless, following hydrolysis, prehydrolysis pH values increased compared to initial pH prior to pretreatment. For instance, as presented in Fig. 2b, the pH value increased up to 0.5 pH units after the hydrolysis while adding 0.25 wt% [Fe^3+^] into the reaction media. In general, a low pH will drive the pretreatment and the ash buffering capacity will mitigate the reduction in pH that results from addition of acid. Notably, some minerals such as KCl, K_2_HPO_4_, and K_2_SO_4_ in biomass could realize the pH-mediated abirritation [41]. In a separate study, Wu et al. [27] reported that pH decreased from 5.7 to 4.5 after autohydrolysis of waste wheat straw while increasing concentrations of FeCl_3_ (0–20 mM), suggesting the buffering capacity of wood poplar is stronger than that of agricultural plants, which needs further investigation. Aqueous solutions of Fe^3+^-HAc (0.25 wt% [Fe^3+^]) are acidic (pH 1.3). To assess whether or not the xylan conversion was dominated by the acidity of Brønsted acid, control experiments using acid to adjust pH in aqueous HAc with an identical proton concentration were implemented. Results show that the potential xylan (91.1%) with Fe^3+^-HAc was significantly higher than that with pH-adjusted HAc (61.6%) at the same acidity. Consistently, higher xylose (73.9%) in the case of Fe^3+^ was obtained compared with pH-adjusted solution (31.8%). This illustrates the activity of Fe^3+^ in xylan conversion (especially xylose) was not solely governed by its Brønsted acidity. In a separate study, Marcotullio and De Jong [42] also reported that at the same pH aqueous FeCl_3_ was remarkably more effective than a strong acid solution. During the experiment, it was hypothesized that the co-existence of FeCl_3_ and HAc would take shape an insoluble ferric acetate compound. However, no precipitate was formed but the green liquid was observed during the xylose production in Fe^3+^-HAc media. It was proposed that the low concentration of FeCl_3_ and HAc may be problematic. Similarly, Zhang et al. [2] reported that adding a comparable amount of HAc into 40% FeCl_3_ solution also resulted in clear liquid, given the assumption that the presence of Cl^−^ and the deficiency of water in FeCl_3_ solution were not positive for the formation of the ferric acetate compound.

### Characteristics of hydrolysis Fe^3+^-HAc-residues

Transition metal species have displayed superior performance on biomass hydrolysis. In this section, the catalytic characteristics of Fe^3+^ on poplar sawdust hydrolysis in HAc reaction media were examined. Herein, several representative conditions (0.04 wt% Fe^3+^, 0.04, 0.17, 0.25, and 0.33 wt% Fe^3+^-6.5% HAc) were selected for the analysis. As a baseline, hydrolysis of poplar catalyzed by 0.04 wt% of Fe^3+^ without HAc was conducted. Table 1 presents the performances of poplar hydrolysis in varying conditions, including the fate of xylan (remaining xylan and xylan loss), composition of pretreated biomass and weight loss.

**Table 1:** Influence of different concentrations of Fe^3+^-assisted HAc media on characteristics of hydrolysis residue

Fe^3+^ (wt%)	HAc	Remaining glucan (%)	Remaining lignin (%)	Remaining xylan (%)	Xylan loss (%)	Weight loss (%)
0.04	No	81.3 ± 1.5	94.1 ± 2.5	39.5 ± 2.4	34.9 ± 2.3	31.1 ± 2.4
0.04	Yes	80.3 ± 2.4	68.1 ± 2.6	19.9 ± 1.6	12.5 ± 2.6	41.2 ± 1.8
0.17	Yes	69.9 ± 2.6	61.4 ± 1.9	7.3 ± 1.6	6.5 ± 1.9	51.5 ± 1.6
0.25	Yes	64.5 ± 1.9	57.5 ± 2.0	0	8.9 ± 2.2	55.4 ± 2.5
0.33	Yes	52.6 ± 3.2	44.5 ± 2.9	0	20.2 ± 2.7	63.8 ± 3.5

Except for the conditions explained in the table, others were kept the same. 2.5 g of poplar sawdust was mixed with 25 g of aqueous reaction media. The concentration of HAc solution was 6.5%, and the reaction temperature was maintained at 170 °C

As shown in Table 1, the remaining xylan and glucan were 39.5% and 81.3%, respectively. Furthermore, the lignin removal efficiency was 5.9%, suggesting the weak catalytic effect of metal (0.04% Fe^3+^) ion in lower lignin removal. However, removal of xylan increased from 60.5 to 80.1% in presence of co-catalysis of 0.04 wt% Fe^3+^ and HAc solution. Simultaneously, the remaining glucan and lignin decreased from 81.3% and 94.1% to 80.3% and 68.1%, respectively. These results indicate that HAc exhibit superior delignification than cellulose removal for poplar, which is consistent with the previous literature [39]. The role of Fe^3+^ dramatically enhanced glucan degradation (Table 1). For instance, the remaining lignin decreased from 68.1 to 44.5%, while the remaining glucan decreased from 80.3 to 52.6% as [Fe^3+^] increased from 0.04 to 0.33 wt%. Mao et al. [14] also reported that Fe^3+^ was more effective in cellulose degradation than that of delignification. As well, the xylan loss and weight loss were obviously affected by the loading of Fe^3+^. From Table 1, the xylan loss firstly decreased from 12.5% to 6–9% as [Fe^3+^] increased from 0.04 to 0.25 wt% and then increased to 20.2% at 0.33 wt% of Fe^3+^. The reason for the increase of xylan loss at 0.33 wt% might lie in the fact that side reactions of furfural were promoted by excessive Fe^3+^, such as self-resinification and condensation of intermediates with furfural. While, weight loss continuously increased from 41.2 to 63.8% as [Fe^3+^] increased from 0.04 to 0.33 wt%. Specifically, it resulted in 100% xylan removal, 64.5% glucan retention and 42.5% delignification in the 0.25 wt% Fe^3+^-HAc-pretreated residue. Taken together, it was exemplified that Fe^3+^-assisted HAc hydrolysis is effective on the catalytic formation of xylose, xylan degradation, glucan recovery, and delignification.

### Enzymatic hydrolysis efficiency of pretreated poplar

**Fig. 3:**
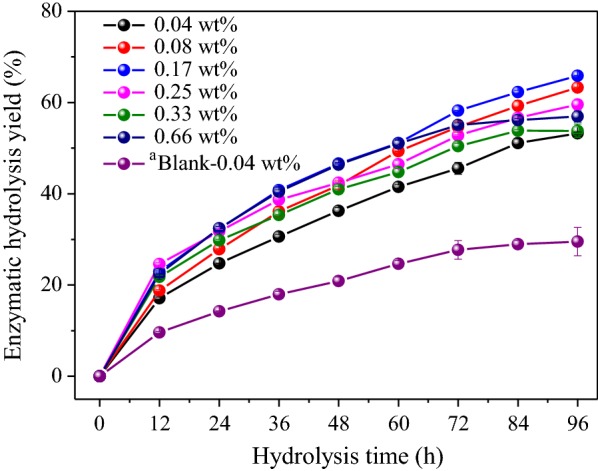
Enzymatic hydrolysis of pretreated poplar xylan at different pretreated conditions for 96 h: different concentrations of Fe^3+^ in aqueous HAc media. ^a^Hydrolysis by 0.04 wt% Fe^3+^ without HAc

For evaluation of the synergistic effects of Fe^3+^ and HAc on poplar sugar generation, pretreated residues underwent enzymatic saccharification. As shown in Fig. 3, the sugar yield reached 31.7% after hydrolysis for 96 h at 0.04 wt% Fe^3+^ in absence of HAc. Huang et al. [11] achieved 23.7% saccharification yield from autohydrolyzed-poplar. Clearly, addition of Fe^3+^ during pretreatment resulted in 33.8% improvement in saccharification efficiency. Additionally, the sugar yield also increased to 53.5% after 96 h at 0.04 wt% Fe^3+^ with HAc, indicating that the synergistic interaction between these two catalysts played an essential role in enzymatic hydrolysis. From Fig. 3, as the Fe^3+^ concentration increased from 0.04 to 0.17 wt%, the sugar yield increased from 53.5 to 65.9%. Results obtained can be attributed to a higher concentration of Fe^3+^ resulting in stimulating release of reactive H_3_O^+^ and facilitating the breaking of “blocking effect” of xylan in the lignocellulosic matrix [43–45]. However, further increasing the Fe^3+^ concentration to 0.66 wt% resulted in a decrease of sugar yield to 56.1% (Fig. 3). Chen et al. [46] also reported that a significant decrease of glucose conversion and a modest increase of cellobiose amount in saccharification was observed when the sulfuric acid concentration was increased from 3 to 15% for the pretreated rice straw. The phenomenon can be ascribed to the inhibitory effect on β-glucosidase led by lignin, and the greater hydrophobicity of pretreated biomass resulting in the enhanced adsorption of enzymes cocktails onto the lignin droplets at high acid concentration [46, 47]. Additionally, xylan degradation yields at 0.04 wt% and 0.17 wt% Fe^3+^-HAc-residues were 80.1% and 92.7%, providing further evidence for the increasing enzymatic efficiency.

As mentioned above, delignification and xylan removal increase the efficacy of enzymatic hydrolysis. Significantly improved sugar conversion (more than 65%) could be achieved at the concentration of 0.17 wt% Fe^3+^ in HAc media. However, the 108 h enzymatic hydrolysis efficiency of HAc-catalyzed poplar was only 51.0% [11]. Overall, for 0.17 and 0.25 wt% Fe^3+^ inclusion into HAc hydrolysis resulted in 72.5% and 73.9% XHR, and 65.9% and 60.3% saccharification efficiency, respectively.

### Fractionation of biomass streams and mass balances

**Fig. 4:**
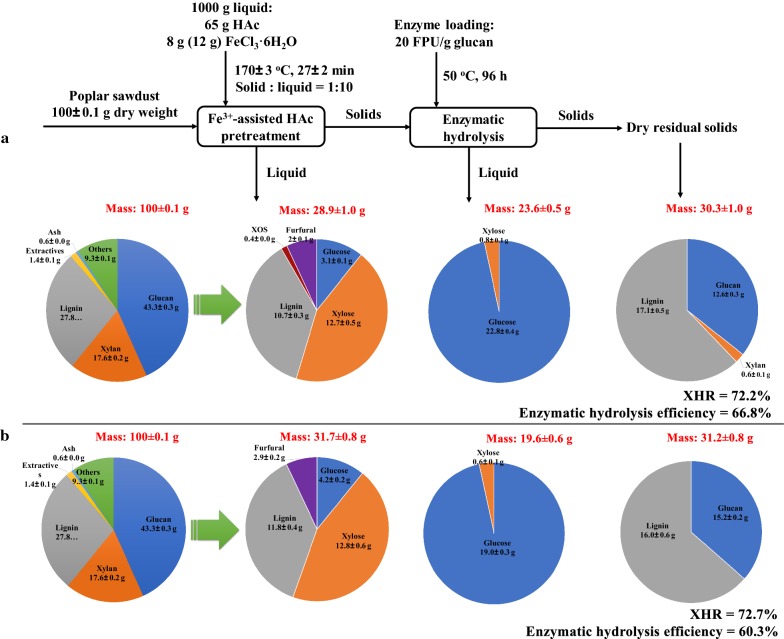
Mass balances for Fe^3+^-assisted HAc pretreatment and saccharification: **a** 0.17 wt% Fe^3+^, **b** 0.25 wt% Fe^3+^

The comprehensive data set of this research can provide the mass flow of poplar biomass components during Fe^3+^-assisted HAc pretreatment and subsequent enzymatic hydrolysis. Mass balances for glucan, xylan, and lignin at two different concentrations of Fe^3+^ (0.17 and 0.25 wt%) were tracked, which may give practical advice and feedback about process validity. As observed in Fig. 4, starting with 100 g poplar, the vast majority of the xylan was extracted and degraded into soluble xylose in the liquid stream after Fe^3+^-HAc pretreatment. As well, up to 40% of the lignin was solubilized in the liquid fraction, while only a small portion of glucan was converted into dissolved glucose, the majority being retained in the pretreated solids and subjected to enzymatic hydrolysis for fermentable sugars production. Figure 4a illustrated that a total amount of 12.7 g of xylose and 22.8 g of glucose were harvested from liquid streams during the whole process of 0.17 wt% Fe^3+^-HAc hydrolysis coupled with enzymatic saccharification, which correspond to the yields of 72.2% and 66.8% for XHR and enzymatic efficiency. At the concentration of 0.25 wt% Fe^3+^ in HAc media, the overall recoveries were 72.7% and 60.3% for XHR and saccharification, respectively (Fig. 4b). Considering the maximum xylose yield, we select the point of 0.25 wt% Fe^3+^ in HAc media for further verification experiment. Taken together, mass balance of poplar biomass illustrates the efficacy of Fe^3+^-assisted HAc pretreatment on lignin carbohydrate complex for production of fermentable sugars.

### Evaluation of different chloride-based salts on poplar xylan degradation in aqueous HAc media

**Fig. 5:**
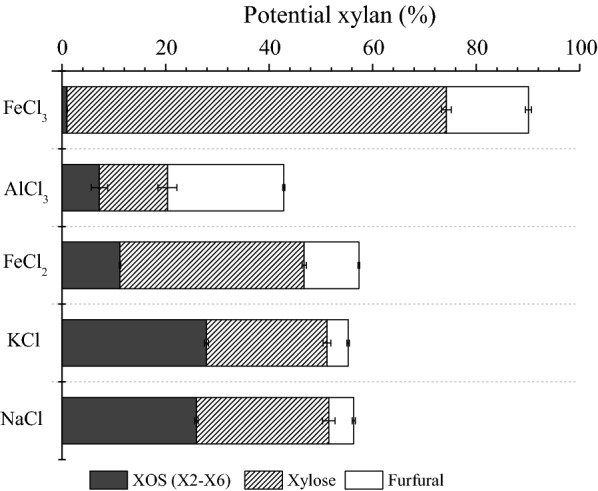
Effects of different chloride-based salts on production of XOS (X2–X6), xylose and furfural in aqueous HAc media

To evaluate the contribution of chloride ion to the catalytic hydrolysis, a comparison of different chloride-based salts for the depolymerization of poplar xylan was also carried out. Encouraged by the optimum concentration (0.25 wt% Fe^3+^, 0.47 wt% Cl^−^) for xylose production, further the same concentration of Cl^−^ was considered for other salts (Fig. 5). As shown in Fig. 5, KCl and NaCl showed similar performance on the release of XOS, xylose and furfural, and only ~ 24% of xylose could be obtained, which suggests that significant amounts of xylan are in the form of xylo-oligomers. At the concentration of 0.47 wt% Cl^−^, the FeCl_2_ dosage or reaction temperature seemed not adequate to fully depolymerize wood xylan, and the yield of potential xylan was detected below 60%. As a member of transition metals, AlCl_3_ was effective on the conversion of xylose to furfural (22.6%). However, a yield of 8.3% XOS still remained in the hydrolysate, which indicates that the pretreatment process may require lower temperature (< 170 °C) for the decrease of furfural production, but longer reaction time (> 27 min) for the effective conversion of XOS to xylose. When comparing different chloride-based salts, FeCl_3_ exhibited the greatest desired effect on xylose production. These data demonstrate that Fe^3+^ rather than Cl^−^ that plays the dominant role in the catalysis enhancement to assist the hydrolysis of HAc.

### Mechanism of co-effects of HAc and Fe^3+^ on xylose production

The pretreatment process for xylose production from poplar xylan involves several complicated processes, including swelling of poplar particles, liberation of acetylated and formylated xylose oligomers. Followed by, formation of precursor (xylose oligomers) of xylose and byproducts, acid-catalyzed formation of xylose and partial xylose degradation to furfural [14]. HAc and FeCl_3_ could impact the conversion process. Therefore, additional tests were conducted to understand the behaviors of these elements and find out catalytic effects on the hydrolysis process.

To understand the effect of catalysts on the hydrolysis pathway from wood xylan to xylose and furfural by Fe^3+^-HAc, beechwood xylan and xylose were treated under the same condition as poplar sawdust fractionation (Table 2). For instance, when xylan was treated with 0.08 wt% of pure Fe^3+^ (Table 2), both xylose (3.1 g/L) and furfural (0.5 g/L) can be stripped out, indicating that certain amounts of Fe^3+^ can realize the further conversion of xylose to furfural. Addition of 6.5% HAc led to a significant increase in furfural generation (from 0.5 to 8.3 g/L, 17-fold), as well as xylose (from 3.1 to 20.1 g/L, sixfold). This effect suggests that HAc displays strong catalytic performance on the formation of furfural. However, Yemis and Mazza [48] found that HAc yielded the lowest furfural production derived from xylan compared to strong mineral acids and other two organic weak acids (phosphoric acid and formic acid). The reason was expected to be different catalytic effect caused by varied process conditions (temperature, solid: liquid ratio and reaction time). When increasing [Fe^3+^] (from 0.08 to 0.25 wt%), it was seen that the concentrations of xylose (from 20.1 to 23.5 g/L) and furfural (from 8.3 to 13.7 g/L) were increased. It was worthwhile to mention that the formation rate of xylose is lower at higher concentrations of Fe^3+^ (from 0.08 to 0.25 wt%), indicating the faster degradation of xylose to furfural.

**Table 2:** Catalytic hydrolysis of beechwood xylan and xylose in various conditions

Reactant	Fe^3+^ (wt%)	HAc (%)	Product concentration (g/L)	
			Xylose	Furfural
Xylan	0.08	No	3.1 ± 0.5^a^	0.5 ± 0.1
	No	6.5	13.9 ± 0.3^a^	5.9 ± 0.4
	0.08	6.5	20.1 ± 0.9^a^	8.3 ± 0.2
	0.25	6.5	23.5 ± 1.3^a^	13.7 ± 0.7
Xylose	0.08	No	59.3 ± 2.6^b^	13.1 ± 0.8
	No	6.5	70.5 ± 0.8^b^	8.7 ± 0.3
	0.08	6.5	54.3 ± 1.84^b^	14.5 ± 0.4
	0.25	6.5	19.8 ± 0.6^b^	22.9 ± 1.0

All the experiments were conducted at 170 °C for 27 min, and the initial concentration of xylan or xylose is 100 g/L

^a^Amounts of xylose production in the media

^b^Amounts of xylose remained in the media

To confirm the above results, further xylose degradation experiments were conducted. When xylose was treated with 0.08 wt% of pure Fe^3+^ (Table 2), a comparatively higher amount of furfural was detected thus verifying that direct catalysis of xylose degradation via Fe^3+^ was easier and more efficient than of xylan. According to Yang et al. [49] a higher furfural yield from xylose than with pure xylan could be attributed to interference from other components in xylan. Addition of 6.5% HAc (Table 2) seemed to contribute less to the hydrolysis of xylose and the formation of furfural compared with those catalyzed solely by Fe^3+^. Likewise, hydrolysis of xylose with HAc alone (Table 2) resulted in low xylose degradation and hence, low furfural production. As shown in Table 2, with the increasing concentration of Fe^3+^ from 0.08 to 0.25 wt% in xylose hydrolysis, remaining xylose concentration decreased from 54.3 to 19.8 g/L and furfural concentration increased from 14.5 to 22.9 g/L. The results indicate that higher catalyst loading of Fe^3+^ is beneficial to facilitate the conversion of xylose to furfural, consistent with the results obtained from the hydrolysis of xylan. With respect to reaction rate, the rate of xylose decomposition was higher than that of furfural formation, suggesting that the co-catalysis of Fe^3+^-HAc significantly accelerated the xylose degradation, whose effect was greater than that of furfural production. It means that the degraded products of xylose contain other value-added chemicals, such as aldehydes, dihydroxyacetone, acids, etc. [50]. In this experiment, decreasing furfural yield was not observed because the reaction time was not long enough for the occurrence of side reactions. In the opinions of Yemis and Mazza [48], a higher concentration of xylose and the presence of furfural would collide with each other, leading to a higher char yield. Additionally, a larger concentration of xylose was susceptible to undertake loss reactions, including successive condensation reactions with furfural and intermediates of xylose transformation [51].

## Conclusions

Fe^3+^-assisted HAc hydrolysis of poplar xylan not only promoted XHR, but also enhanced the xylan degradation, delignification and glucan release. An overall process for XHR coupled with enzymatic hydrolysis was developed. Optimized conditions obtained the xylose yields of 72.5% and 73.9% at concentrations of 0.17 wt% and 0.25 wt% [Fe^3+^] in HAc media. For enzymatic hydrolysis, a significantly improved sugar conversion (from 51.0 to 65.9%) was achieved compared with HAc alone. Catalytic mechanisms were verified by employing model compounds of xylan and xylose. This study provides a deep understanding of the metal–organic hydrolytic conversion of wood polysaccharides to valuable products.

## Methods

### Material

A fast-growing poplar tree (3 years old) was harvested from the Jiangsu province in China. The wood sample was ground to pass through a 20-mesh size screen (< 0.85 mm), air-dried, homogenized in a single lot, and stored at room temperature. The moisture content of milled sawdust was approximately 10% based on total wet biomass weight. The composition of the poplar sawdust is glucan (43.3%), xylan (17.6%), acid-insoluble lignin (3.7%), acid-soluble lignin (24.2%), and ash content (0.6%) by dry weight. HAc, FeCl_3_·6H_2_O, FeSO_4_·7H_2_O, Fe_3_O_4_, sulfuric acid and xylan (from beechwood, > 90% xylose residues) used in the experiments were analytical reagents without further purification. Xylose, xylobiose (X2), xylotriose (X3), xylotetraose (X4), xylopentaose (X5), and xylohexaose (X6) were purchased from Shanghai Yikuo Instrument Co., Ltd. (Shanghai, China), and were used as standards.

### Experimental procedure for conversion

A series of experiments were carried out in a sealed 30 mL stainless steel tube reactor using diluted HAc as the catalyst in presence of different species of Fe ions. The biomass was thoroughly mixed with a certain amount of HAc and varying concentrations of Fe species in the digester and then pre-impregnated for 1 h at room temperature. Based on our previous optimization, the reaction temperature, time and HAc concentration were chosen to be 170 °C, 27 min and 6.5% (v/v), respectively [11]. In each experiment, the reactor was loaded with an amount of feedstock equivalent to 2.5 g of dry matter. To ensure the homogeneous distribution of aqueous solution into the granular poplar, a liquid-to-solid ratio of 10:1 was used. After the pretreatment, the solid was thoroughly washed with water to remove the residual catalysts.

Following pretreatment, enzymatic hydrolysis was carried out at 5% (w/v) for 96 h. The recovered solids were mixed with 50 mM sodium citrate buffer at pH 4.8, 0.2% (w/v) tetracycline, and the cellulose enzyme (C2730, Celluclast^®^ 1.5 L, Novozymes Inc.). The enzyme loading was 20 FPU/g glucan at 50 °C and 150 rpm in an orbital shaker (New Brunswick) [11]. Samples were taken periodically from the reaction mixture at 12 h intervals and centrifuged at 10,000 rpm for 5 min to detect the released glucose and cellobiose. For the quantification of fermentable sugars during enzymatic hydrolysis, the supernatants in the samples were diluted and followed by filtration through a 0.22 μm syringe filter prior to high-performance liquid chromatography (HPLC) analysis.

### Analytical methods and calculations

The chemical compositions of all the material were determined according to the standard laboratory analytical procedures developed by the National Renewable Energy Laboratory [52]. The moisture content of the sample was measured by an infrared moisture meter (FD-720, KETT). Monosaccharides and furfural were analyzed by HPLC (Agilent 1260 series, Agilent Technologies, Santa Clara, CA, USA) equipped with a refractive index detector and Aminex Bio-Rad HPX-87H column using 0.05 M H_2_SO_4_ at a flow rate of 0.6 mL/min and column temperature of 55 °C. Xylooligosaccharides were determined using an HPAEC-PAD (Dionex ICS-5000) equipped with a carbopac PA-200 as the anion-exchange column and the separation method used herein was reported elsewhere [53]. Briefly, separation was achieved via gradient elution using mobile phase of 0.1 M NaOH and 0.5 M NaOAc containing 0.1 M NaOH.

The xylose, XOS, furfural yield, enzymatic efficiency, remaining glucan and xylan, xylan loss and weight loss were calculated according to the following equations.

$${\text{Xylose }}\;{\text{yield}}\,\left( \% \right) = \frac{{{\text{xylose}} \left[ {\text{g}} \right] {\text{in}}\;{\text{supernatants}}}}{{{\text{xylan}} \left[ {\text{g}} \right] {\text{in }}\;{\text{raw}}\;{\text{material}}}} \times 100$$

$${\text{Furfural}}\; {\text{yield}}\,\left( \% \right) = \frac{{{\text{furfural}} \left[ {\text{g}} \right] {\text{in}}\;{\text{supernatants}}}}{{{\text{xylan}} \left[ {\text{g}} \right]{\text{in}}\; {\text{raw}}\;{\text{material}} }} \times 100$$

$${\text{XOS}}\; {\text{yield}}\,\left( \% \right) = \frac{{{\text{sum}}\;{\text{of}}\;{\text{all}}\;{\text{XOS}}\; {\text{in}}\;{\text{supernatants }}\left( {{\text{DP }}2 - 6} \right)\left[ {\text{g}} \right]}}{{{\text{xylan}} \left[ {\text{g}} \right] \,{\text{in}}\;{\text{raw}}\;{\text{material}} }} \times 100$$

$${\text{Enzymatic}}\;{\text{efficiency}}\,\left( \% \right) = \frac{{\left( {{\text{glucose}} + {\text{cellobiose}} \times 1.05} \right)\left[ {\text{g}} \right] \times 0.9}}{{{\text{glucan}} \left[ {\text{g}} \right] \, {\text{in}}\;{\text{pretreated}}\;{\text{solids}} }} \times 100$$

$${\text{Remaining}}\;{\text{glucan}} = \frac{{{\text{glucan }}\left[ {\text{g}} \right] \,{\text{in}}\;{\text{residue}}}}{{{\text{glucan}} \left[ {\text{g}} \right] \, {\text{in}}\;{\text{raw}}\;{\text{material}}}} \times 100\%$$

$${\text{Remaining xylan}} = \frac{{{\text{xylan }}\left[ {\text{g}} \right] \, {\text{in residue}}}}{{{\text{xylan }}\left[ {\text{g}} \right] \,{\text{in raw material}}}} \times 100\%$$

$${\text{Potential}}\;{\text{xylan}} = \left( {{\text{xylose}}\;{\text{yield}} + {\text{furufral}}\;{\text{yield}} + {\text{XOS}}\;{\text{yield}}} \right) \times 100\%$$

$${\text{Xylan}}\;{\text{loss}} = \left( {1 - {\text{remaining}}\;{\text{xylan}} - {\text{potential}}\;{\text{xylan}}} \right) \times 100\%$$

$${\text{Weight}}\;{\text{loss}} = \left( {1 - \frac{{{\text{mass}}\;{\text{of}}\;{\text{dried residue}} \, \left[ {\text{g}} \right]}}\,{{{\text{raw}}\;{\text{material}}\,\left[ {\text{g}} \right]}}} \right) \times 100\% .$$

## Data Availability

All data generated and analyzed in this study are included in this published article.

## Abbreviations

HAc: Acetic acid
XHR: Xylose-hydrolysate recovery
XOS: Xylooligosaccharides
X2: Xylobiose
X3: Xylotriose
X4: Xylotetraose
X5: Xylopentaose
X6: Xylohexaose
